# Impact of Co‐Existing Subjective Cognitive Decline and Physical Frailty on Subsequent Cognitive Impairment and Dementia in Older Adults

**DOI:** 10.1002/alz70860_107312

**Published:** 2025-12-23

**Authors:** Jing Huang, Qian‐Li Xue, Sarah L Szanton, Junxin Li

**Affiliations:** ^1^ Johns Hopkins University, Baltimore, MD, USA; ^2^ Johns Hopkins University School of Medicine, Baltimore, MD, USA

## Abstract

**Background:**

Subjective cognitive decline (SCD), characterized as self‐reported decline in cognitive capability without clinical cognitive impairment, and physical prefrailty/frailty are increasingly recognized as independent risk factors for subsequent cognitive impairment and dementia. Although these two conditions frequently coexist in older adults, it remains unclear whether their co‐occurrence poses a greater risk for cognitive impairment and dementia compared to either condition alone. This study aims to examine whether older adults with co‐existing SCD and physical frailty experience a higher incidence of cognitive impairment and dementia over a 10‐year follow‐up period, compared to those with neither condition or only one of the conditions.

**Method:**

Using six waves of data from the Health and Retirement Study (HRS) from 2010 to 2020, we included 4,643 older adults aged ≥65 years who had physical frailty and cognitive data, without cognitive impairment at baseline. Participants were categorized into four groups: co‐existing SCD and pre‐frailty/frailty, SCD only, pre‐frailty/frailty only, and healthy. The incidence of cognitive impairment and dementia was assessed using validated cutoffs from the HRS cognitive test and proxy‐reported cognitive status. Discrete‐time survival analysis was employed to examine the association between group membership and the incidence of cognitive impairment and dementia, adjusting for sociodemographic and health‐related covariates.

**Result:**

Of the sample, 714 (15.4%) had co‐existing SCD and physical prefrailty/frailty, 427 (9.2%) had SCD only, 1,904 (41%) had pre‐frailty/frailty only, and 1,598 (34.4%) were classified as healthy. Individuals with co‐existing SCD and physical frailty had significantly higher risks of cognitive impairment compared to healthy individuals (OR: 1.62, 95% CI: 1.39–1.89), those with SCD only (OR: 1.34, 95% CI: 1.09–1.64), and those with pre‐frailty/frailty only (OR: 1.21, 95% CI: 1.05–1.39). Similarly, their risk of dementia was significantly higher compared to healthy individuals (OR: 1.99, 95% CI: 1.49–2.68), those with SCD only (OR: 1.63, 95% CI: 1.10–2.42), and those with pre‐frailty/frailty only (OR: 1.41, 95% CI: 1.11–1.81).

**Conclusion:**

Co‐existing SCD and physical pre‐frailty/frailty are associated with increased risks of cognitive impairment and dementia compared to either condition alone. This helps to identify the at‐risk groups and enable targeted early intervention to help prevent dementia.

